# Antimicrobial resistance and the presence of extended-spectrum beta-lactamase genes in *Escherichia coli* isolated from the environment of horse riding centers

**DOI:** 10.1007/s11356-018-2274-x

**Published:** 2018-05-23

**Authors:** Katarzyna Wolny-Koładka, Anna Lenart-Boroń

**Affiliations:** Department of Microbiology, University of Agriculture in Cracow, Mickiewicza Ave 24/28, 30-059 Cracow, Poland

**Keywords:** Antibiotics, Antimicrobial resistance, *Escherichia coli*, Extended-spectrum beta-lactamases, horses

## Abstract

The aim of the study was to determine the antimicrobial resistance profile and the occurrence of extended-spectrum beta-lactamase genes and to analyze the genetic diversity of *Escherichia coli* strains isolated from the environment of horse riding centers. The study was conducted using *E. coli* strains isolated from the air, manure, and horse nostril swabs in three horse riding centers differing in the system of horse keeping—stable (OJK Pegaz and KJK Szary) and free-range (SKH Nielepice). Resistance to antibiotics was determined using the disk-diffusion method, and the PCR technique was employed to detect the extended-spectrum β-lactamase (ESBL) genes, while the genetic diversity of strains was assessed by rep-PCR. A total of 200 strains were collected during the 2-year study, with the majority isolated from KJK Szary, while the smallest number was obtained from SKH Nielepice. The strains were mostly resistant to ampicillin, aztreonam, and ticarcillin. The tested strains were most frequently resistant to one or two antibiotics, with a maximum of ten antimicrobials at the same time. Two multidrug-resistant (MDR) strains were detected in OJK Pegaz while in KJK Szary there were two MDR and one extensively drug-resistant (XDR) strain. The ESBL mechanism was most frequently observed in OJK Pegaz (20.31% of strains) followed by KJK Szary (15.53% of strains) and SKH Nielepice (15.15% of strains). Among the ESBL-determining genes, only *blaTEM* and *blaCTXM-9* were detected—*blaTEM* was mostly found in KJK Szary (53.40% of strains), while the second detected gene—*blaCTXM-9*—was most frequent in SKH Nielepice (6.06% of strains). The rep-PCR genotyping showed high variation among the analyzed strains, whereas its degree differed between the studied facilities, indicating that the type of horse keeping (stable vs. free-range) affects the genetic diversity of the *E. coli* strains. Having regard to the fact that the tested strains of *E. coli* were derived from non-hospitalized horses that were not treated pharmacologically, we can assume that the observed antimicrobial resistance may be of both—natural origin, i.e., not the result of the selection pressure, and acquired, the source of which could be people present in the horse riding facilities, the remaining horses which were not included in the study, and air, as well as water, fodder, and litter of the animals. Therefore, it can be concluded that the studied horses are the source of resistant *E. coli* and it is reasonable to continue monitoring the changes in antimicrobial resistance in those bacteria.

## Introduction

Bacterial resistance to antimicrobial agents is an increasing and globally occurring problem; therefore, monitoring this phenomenon and understanding its molecular basis is extremely important. Obtaining information about pathways of spreading of antimicrobial resistance-determining genes and their transmission between various components of the ecosystem will contribute to the development of new concepts to counteract this process (Angulo et al. 2004; Nunnery et al. 2006; Kadlec and Schwarz 2008). Horses are a natural reservoir of antibiotic-resistant microorganisms, which has direct effect on their health, treatment efficiency, and epidemiological safety of people who get in contact with these animals. Moreover, transmission of zoonotic, bacterial risk factors poses a serious threat to public health (Ahmed et al. 2010). The frequent use of similar antibiotics in the treatment of humans and horses is also a major problem, which makes it difficult to find an effective pharmacological agent in case of infection. In addition, *Escherichia coli* is a commensal, opportunistic pathogen, constantly present both in the mammalian digestive tract and in the environment, e.g., in water or soil. This contributes to the transfer of genes between strains and to the increase in the drug resistance in environmental bacteria (Alekshun and Levy 2007; Scott 2008).

There are reports in the world literature on the spread of drug resistance genes in *E. coli* isolated from animals, including horses (Sáenz et al. 2004; Dunowska et al. 2006; Vo et al. 2007; Ahmed et al. 2010). Unfortunately, despite the fact that in Poland horse breeding has a long tradition and horse riding is becoming increasingly popular, there is still a lack of research regarding this issue (Wolny-Koładka and Malina 2017). People using this form of leisure, through direct contact with animals, are clearly exposed to zoonotic risk factors, including drug-resistant bacteria (Ahmed et al. 2010). It should also be remembered that horses have the status of both accompanying and slaughter animals, and Poland is among countries which produce the largest amounts of horse meat and export live horses for slaughter to—among others—Italy, France, or Belgium. A breeder who owns horses for slaughter is obliged to keep detailed documentation confirming the health condition of animals and a list of prescribed medicines including antibiotics (Commission Regulation 2008). Veterinarians report in detail the medicinal methods used and drugs administered to slaughter horses; they are also required to keep records of animals that lost the status of slaughter animals. Such procedures are intended to minimize the risk of improper quality horse meat on the food market, which would be dangerous to consumers’ health. A serious problem arises while treating a horse not for slaughter, when the veterinarian can arbitrarily choose a medicine, based on the list of pharmaceuticals registered for treating horses, or in justified cases—outside of the list. Also, information about the method of treatment does not have to be recorded in the medical documentation of such horse. This is the case of sport horses and horses used in recreation and agriculture, as they are usually presented as animals excluded from slaughter. Considering the fact that after several years these horses are traded and go to slaughter without proper veterinary documentation, they pose a serious threat to food safety. It turns out that in the post-slaughter collected meat samples, there may be residues of drugs. In addition, a significant problem is the falsification of meat products from animals belonging to other species by horse meat of unknown origin. Such activity is obviously illegal, and offered meat can endanger the health and life of consumers (Bryan et al. 2010; Wróblewski and Wojtaszek 2015). Apart from the risk associated with the consumption of meat from horses that could have been treated with antibiotics, the use of inappropriately processed manure derived from these animals as fertilizers is also problematic. In that case, we are dealing with environmental contamination by pathogenic microorganisms, often highly resistant to antibiotics, and with introduction of antibiotics and their metabolites into water and soil (Venglovsky et al. 2009).

One of the most common mechanisms of resistance in *E. coli* is their ability to produce extended-spectrum beta-lactamases (ESBL). The presence of *Salmonella enterica*, *E. coli*, and *Klebsiella pneumoniae* beta-lactamase-producing strains has already been detected in feces of pigs, cattle, and horses (Wellington et al. 2013). Strains, in which the ESBL mechanism has been found, are very dangerous from an epidemiological point of view, because they hydrolyze all penicillins, cephalosporins, and monobactams. In addition, they may exhibit cross-resistance to trimethoprim/sulfamethoxazole and quinolones (Picozzi et al. 2014). The extended-spectrum β-lactamase (ESBL)-encoding genes are rapidly spreading, also among strains of different species, which is due to their location on conjugation plasmids (Marcade et al. 2009; Rawat and Nair 2010). Hence, the high pathogenicity and antimicrobial resistance of ESBL-producing *E. coli* strains. Moreover, these isolates can be donors of resistance genes to many commonly used antibiotics, which hinders rational antibiotic therapy. This is why it is so important to understand and monitor the phenomenon of antimicrobial resistance in the environment including the identification of ESBL-producing strains.

The aim of this study was to assess the genetic diversity of *E. coli* strains isolated from the air, manure, and nostrils of horses from three horse riding centers that differ in the horse keeping system—stable and free-range (non-stable system). The antimicrobial resistance profile was determined, with particular emphasis on the occurrence of the ESBL mechanism. The information provided will help to determine, whether there are multidrug-resistant *E. coli* strains spread in the environment of the horse riding centers, which could pose a threat to public health.

## Materials and methods

### Characteristics of sampling points and sample collection

The samples of air, manure, and nostril swabs were collected every 2 months over the period of 2 years (2015–2016), which gave 12 series of material collection. Horses for the study were selected on the basis of their owners’ declarations that they would not be sold during the study period, and that the animals were kept in good condition and were not continuously treated pharmacologically. In this study, *E. coli* were isolated from the air, manure, and nostrils of horses kept in three horse riding centers, two of which have stables and one is free-range (non-stable). The horse keeping system depends on their race, age, sex, and intended use as well as the possibilities of a given facility. The box system is the most popular model of horse keeping, and it does not require frequent supervision; while being easily accessible to the animals, it is also economically viable. On the other hand, it clearly limits the movement of animals and contact with other individuals. The box system is used in the case of sport horses, noble breeds, or especially valuable individuals, such as foals, stallions, and aggressive individuals. On the other hand, the non-stable system provides the horses with the possibility of unrestricted movement and contact with other individuals, most suited to the nature of the horse. The animals spend most of their time in pasture in large groups, returning to farm buildings in the case of heavy cold or when they are being used. The non-stable system is ideal for Hutsul ponies, rugged, resistant to adverse climatic conditions, and with strong heard instincts (Waran 2002).

The study was conducted in three Lesser Poland (southern Poland) horse riding centers. The Horse Riding Center Pegaz in Kraków (OJK Pegaz) has one small stable, common for all horses, with seven closed and eight boxes opening to the outside. There are 13 horses in the facility, and the remaining 2 are horses owned by private people, not participating in the study. The horses in the center are recreational, and apart from private horses, there is no rotation. The air was collected in five points. Points 1 and 2 were located inside the closed stable, points 3 and 4 in the open box stalls, and point 5 in front of the stable, outdoors (control point). The Horse Riding Club Szary in Michałowice (KJK Szary) is a large and extremely modern center with 100 box stalls and with recreational and sport horses, and the facility also runs a guesthouse for horses, hence the large rotation of animals. The air samples were collected in 10 sites, points 1–9 within the stable and point 10 (control) located outdoors. The sampling sites in OJK Pegaz and KJK Szary were evenly distributed so that the air in the stable could be analyzed in a representative way. In both OJK Pegaz and KJK Szary, 13 horses were selected for the study, so the experiment was planned to collect biological material in the form of swabs from the same horse and at the same time to collect fresh manure from its box. The Hutsul Pony Stud Farm in Nielepice (SKH Nielepice) is the only one that runs the non-stable husbandry. Horses of the Hutsul breed stay in the open air all year round and use only shelters in wooden sheds without doors. The air samples in SKH Nielepice were collected in four points relevant for the operation of the stud (1—roof for riders, 2—saddle room, 3—roof for horses, 4—paddock). In SKH Nielepice, 22 horses were subjected to the analysis, so the experiment was planned to collect the biological material in the form of a swab from the same horse. Due to the non-stable horse keeping, fresh manure was collected from six points located under a roofed shelter where horses await their riders. In all three horse riding centers, manure was collected to 500-ml sterile containers and swabs from horse nostrils were taken by sterile swabs with a transport medium (BTL, Poland) and immediately transported to the laboratory to isolate *E. coli*.

### Identification of *E. coli*

Microorganisms were isolated differently depending on the source: manure—with the serial dilutions method; air—with collision method using MAS-100 air sampler (Merck, Switzerland) according to the manufacturer’s instructions (Operator’s Manual MAS-100™ Professional Microbial Air Monitoring System for the Microbiological Testing of Air n.d.); and horse nostrils—swabs followed by inoculation of the biological material on microbiological medium. Selective, chromogenic medium, TBX agar (Tryptone Bile X-glucuronide agar, BTL, Poland), microscopic observations of Gram-stained smears, and MALDI-TOF MS (Bruker Daltonik, Germany) were used to identify the *E. coli* strains (Bohme et al. 2010; Seng et al. 2010; Croxatto et al. 2012; Kosikowska et al. 2015; Wolny-Koładka and Malina 2017). The aim was to collect a number of *E. coli* isolates from each of the analyzed environments having regard to the fact that the strains may be isolated with different frequency (e.g., *E. coli* is more frequently present in manure than in the air).

### Antimicrobial resistance and detection of ESBL mechanism

The antimicrobial resistance of the collected *E. coli* strains was determined by the disk-diffusion method, recommended by the European Committee on Antimicrobial Susceptibility (EUCAST 2017), using MHA medium (Mueller-Hinton agar, BTL, Poland) and antimicrobial disks (Oxoid, Ireland). For antibiotics not included therein, the recommendations presented by other authors were used, i.e., Kronvall et al. (1984)—cefalotin, Turnidge (2011)—cefazolin, Barry et al. (1983)—cefamandole, and Sader et al. (2007)—tetracycline. The ESBL mechanism was detected with the double-disk synergy test (Drieux et al. 2008). After incubation for 18–24 h at 37 °C, the growth inhibition diameters around the antimicrobial disks were measured (mm) and the results were compared with the breakpoint values recommended by the EUCAST (2017). Quality control was performed using the *E. coli*-type strain ATCC 25922.

### DNA extraction and detection of ESBL-determining genes

Bacterial genomic DNA was extracted from the cultures obtained in the course of the study and from the control *E. coli* strain ATCC 25922 using the Genomic Mini DNA extraction kit (A&A Biotechnology, Poland), following the manufacturer’s instructions. In order to determine the presence of ESBL-determining genes, PCR tests were conducted using specific primers (Table 1): blaCTXM3 (Costa et al. 2006), blaCTXM9 (Simarro et al. 2000), blaOXA, blaSHV, and blaTEM (Sáenz et al. 2004). The reactions were performed in a 25-μl volume containing 50 ng of DNA template, 12.5 pM of each primer, 2.5 mM of dNTP, 1 × PCR buffer, and 1 U DreamTaq DNA polymerase (Thermo Scientific, USA). The following temperature profile was used for the reactions: initial denaturation at 95 °C for 5 min, followed by 35 cycles of 94 °C for 45 s, annealing for 45 s at temperatures corresponding to individual primers, then extension at 72 °C for 1 min with final extension at 72 °C for 10 min and then storage at 4 °C. PCR amplifications were performed in T100 Thermal Cycler (Bio-Rad, USA). The PCR products were electrophoresed for 60 min in 1× TBE, 1% agarose gel, stained with SimplySafe (0.5 mg/ml; EurX, Poland), visualized in UV light, and documented by the Gel Doc system (Bio-Rad, USA).

**Table 1:** Description of primers used in the study

Gene	5′–3′ sequence	Annealing temperature (°C)	Product length (bp)	Reference
*blaCTXM3*	F: GTTACAATGTGTGAGAAGCAG R: CCGTTTCCGCTATTACAAAC	60	800	Costa et al. 2006
*blaCTXM9*	F: GTGACAAAGAGAGTGCAACGG R: ATGATTCTCGCCGCTGAAGCC	54	860	Simarro et al. 2000
*blaOXA*	F: ACACAATACATATCAACTTCGC R: AGTGTGTTTAGAATGGTGATC	61	813	Sáenz et al. 2004
*blaSHV*	F: CACTCAAGGATGTATTGTG R: TTAGCGTTGCCAGTGCTCG	52	885	Sáenz et al. 2004
*blaTEM*	F: ATTCTTGAAGACGAAAGGGC R: ACGCTCAGTGGAACGAAAAC	60	1150	Sáenz et al. 2004

### Molecular differentiation of bacterial strains

Molecular differentiation of *E. coli* strains was based on the rep-PCR conducted using BOXA1R primer (Versalovic et al. 1994). PCR reactions were conducted in duplicates. Each reaction was performed in 25 μl containing approximately 20 ng of DNA template, 12.5 pM of the primer, 2.5 mM of dNTP, 1× PCR buffer, and 1 U of DreamTaq DNA polymerase (Thermo Scientific, USA). The PCR amplification was performed in T100™ Thermal Cycler (Bio-Rad, USA) using the following temperature profile: initial denaturation at 94 °C for 5 min, followed by 25 touchdown cycles of denaturation at 94 °C for 30 s, annealing starting from 67.5 °C with temperature decreasing by 0.5 °C in each cycle until 55 °C for 30 s and elongation at 72 °C for 1 min and then 20 cycles of denaturation at 94 °C for 30 s, annealing at 55 °C for 30 s and elongation at 72 °C for 1 min, and final elongation for 10 min. The PCR products were electrophoresed for 120 min in 1.5% agarose gel, stained with SimplySafe (EurX, Poland) up to 0.5 mg/ml in 1× TBE buffer. After the electrophoresis, the gel was analyzed with UV light and Gel Doc (Applied Biosystems, USA). The resulting BOX-PCR bands were scored on agarose gels—the bands present on both gels were taken into consideration and encoded in the binary matrix.

### Statistical analysis

Statistica v. 12.5 (StatSoft) was used to conduct the chi-square test in order to verify the significance of differences in the resistance to the tested antimicrobial agents in *E. coli* strains isolated from three horse riding centers (OJK Pegaz, KJK Szary, SKH Nielepice).

The presence of clonal strains in the rep-PCR analysis was verified using FaBox (Villesen 2007) by searching for individual haplotypes. Strains carrying the same haplotype were considered clonal. The intra- and inter-population variation was assessed using AMOVA carried out with Arlequin 3.5.2.2. (Excoffier and Lischer 2010) with rep-PCR data encoded as standard data type. The genetic distances between strains were calculated based on the binary matrix of amplified fragments, and unweighted pair group method with arithmetic mean (UPGMA) dendrograms were constructed using SplitsTree 4 (Huson and Bryant 2006).

## Results and discussion

A total of 200 *E. coli* strains were collected in the 2-year study, including 64 from OJK Pegaz, 103 from KJK Szary, and 33 from SKH Nielepice (Table 2). The *E. coli* isolates from swabs and manure were the predominant ones, while airborne ones were the least numerous. Air is not a favorable environment for microbial multiplication and dwelling, but it favors movement of microorganisms (Wolny-Koładka et al. 2014). *E. coli*, occurring mainly in fresh feces, can enter the air with dust particles and in the form of bioaerosol can be inhaled by horses and people in their direct surroundings (Heuer et al. 2011). The presence of fecal bacteria in the air is disturbing and may indicate microbial contamination of the stables. It should be noted that the smallest share among the collected *E. coli* strains had those from SKH Nielepice, and these bacteria were not found in any of the air samples. The reasons for this situation in SKH Nielepice is the free-range horse keeping system, as the horses spend most of the year on pastures. The presented data clearly show that *E. coli* is more frequently isolated in the horse riding centers with the box stall type of horse keeping (OJK Pegaz and KJK Szary), also in the air of those facilities. Furthermore, in OJK Pegaz and KJK Szary, the most numerous of the collected strains were those isolated from the nostril swabs, indicating that the animals spend much time in their box stalls, where they have direct contact with their own feces.

**Table 2:** Frequency (%) of antimicrobial resistance in *E. coli* strains isolated from three horse riding centers

Antimicrobial, symbol (μg)	Breakpoint values (mm)	Strain origin *n* = 200			Mean	OJK Pegaz *n* = 64			Mean	KJK Szary *n* = 103			Mean	SKH Nielepice *n* = 33			Mean
		OJK	KJK	SKH		M	A	S		M	A	S		M	A	S	
Number of isolates		**64**	**103**	**33**	**66.67**	**26**	7	**31**	**21.33**	**39**	**17**	**47**	**34.33**	**19**	0	**14**	**11**
Share % of isolates in the total number of strains		**32**	**51.5**	**16.5**	–	**40.63**	**10.93**	**48.44**	–	**37.87**	**16.5**	**45.63**	–	**57.58**	0	**42.42**	–
Amikacin (AK, 30)	18/15 (EUCAST 2017)	3.13	0	0	1.04	0	0	6.45	2.15	0	0	0	0	0	0	0	0
Amoxicillin/clavulanic acid (AMC, 30)	19 (EUCAST 2017)	1.56	1.94	9.09	4.2	0	**14.29**	0	4.76	0	0	4.26	1.42	0	0	**21.43**	7.14
Ampicillin (AMP, 10)	14 (EUCAST 2017)	**14.06**	2.91	**24.24**	**13.74**	7.69	**14.29**	**19.35**	**13.78**	2.56	5.88	2.13	3.52	5.26	0	**50**	**18.42**
Aztreonam (ATM, 30)	26/21 (EUCAST 2017)	9.38	**15.53**	6.06	**10.32**	**11.54**	**28.57**	3.23	**14.45**	7.69	**23.53**	**19.15**	**16.79**	0	0	**14.29**	4.76
Cefamandole (MA, 30)	18/14 (Barry et al. 1983)	1.56	3.88	0	1.82	0	**14.29**	0	4.76	2.56	5.88	4.26	4.23	0	0	0	0
Cefepime (FEP, 30)	27/21 (EUCAST 2017)	1.56	1.94	0	1.17	3.85	0	0	1.28	0	0	4.26	1.42	0	0	0	0
Cefotaxime (CTX, 30)	20/17 (EUCAST 2017)	0	0.97	0	0.32	0	0	0	0	0	0	2.13	0.71	0	0	0	0
Cefoxitin (FOX, 30)	19 (EUCAST 2017)	3.13	3.88	9.09	5.37	3.85	**14.29**	0	6.04	2.56	0	6.38	2.98	5.26	0	**14.29**	6.52
Ceftazidime (CAZ, 30)	22/19 (EUCAST 2017)	4.69	**12.62**	3.03	6.78	3.85	**14.29**	3.23	7.12	7.69	**11.76**	**17.02**	**12.16**	0	0	7.14	2.38
Cefalotin (KF, 30)	13 (Kronvall et al. 1984)	3.13	5.83	**18.18**	9.04	0	0	6.45	2.15	2.56	0	**10.64**	4.4	5.26	0	**35.71**	**13.66**
Cefazolin (KZ, 30)	23/19 (Turnidge 2011)	0	3.88	9.09	4.32	0	0	0	0	0	0	8.51	2.84	5.26	0	**14.29**	6.52
Ciprofloxacin (CIP, 5)	26/24 (EUCAST 2017)	0	0.97	0	0.32	0	0	0	0	0	0	2.13	0.71	0	0	0	0
Gentamicin (CN, 10)	17/14 (EUCAST 2017)	3.13	1.94	0	1.69	0	0	6.45	2.15	0	5.88	2.13	2.67	0	0	0	0
Netilmicin (NET, 30)	15/12 (EUCAST 2017)	0	1.94	3.03	1.66	0	0	0	0	2.56	0	2.13	1.56	5.26	0	0	1.75
Piperacillin (PRL, 100)	20/17 (EUCAST 2017)	3.13	3.88	0	2.34	0	**14.29**	3.23	5.84	5.13	5.88	2.13	4.38	0	0	0	0
Piperacillin/tazobactam (TZP, 110)	20/17 (EUCAST 2017)	0	1.94	0	0.65	0	0	0	0	2.56	0	2.13	1.56	0	0	0	0
Tetracycline (TE, 30)	15/11 (Sader et al. 2007)	**14.06**	4.85	0	6.31	**11.54**	**14.29**	**16.13**	**13.98**	2.56	5.88	6.38	4.94	0	0	0	0
Ticarcillin (TIC, 75)	23 (EUCAST 2017)	**18.75**	5.83	**21.21**	**15.26**	**15.38**	**14.29**	**22.58**	**17.42**	2.56	5.88	8.51	5.65	5.26	0	**42.86**	**16.04**
Tobramycin (TOB, 10)	17/14 (EUCAST 2017)	3.13	1.94	3.03	2.7	0	0	6.45	2.15	0	0	4.26	1.42	5.26	0	0	1.75
Trimethoprim/sulfamethoxazole (SXT, 25)	14/11 (EUCAST 2017)	1.56	2.91	0	1.49	3.85	0	0	1.28	5.13	5.88	0	3.67	0	0	0	0
ESBL	–	**20.31**	**15.53**	**15.15**	**17**	**23.08**	**42.86**	**12.9**	**26.28**	**15.38**	5.88	**19.15**	**13.47**	**21.05**	0	7.14	9.4
blaTEM	–	**46.88**	**53.4**	**30.3**	**43.53**	**50**	**71.43**	**38.71**	**53.38**	**43.59**	**76.47**	**53.19**	**57.75**	**31.58**	0	**28.57**	**20.05**
blaCTXM-9	–	4.69	0.97	6.06	3.91	3.85	**14.29**	3.23	7.12	0	5.88	0	1.96	5.26	0	7.14	4.13

Values > 10 are bolded

*M* manure, *A* air, *S* swab

Disk-diffusion tests allowed to determine the antimicrobial resistance of the analyzed *E. coli* strains, and the detailed results are shown in Table 2. The bacteria were most frequently resistant to ampicillin and ticarcillin (respectively OJK Pegaz—14.06 and 18.75%, SKH Nielepice—24.24 and 21.21%), aztreonam (15.53%) and ceftazidime (12.62%) (KJK Szary), cefalotin (18.18%—SKH Nielepice), and tetracycline (14.06%—OJK Pegaz). In OJK Pegaz, the *E. coli* strains collected from all three environments (manure, air, and nostrils) showed increased resistance to tetracycline (respectively 11.54, 14.29, and 16.13%) and ticarcillin (15.38, 14.29, and 22.58%). All isolates were, however, susceptible to cefotaxime, cefazolin, ciprofloxacin, netilmicin, and piperacillin/tazobactam. The percentage share of strains resistant to at least one or more tested antimicrobial agents in different environments was as follows: 50% manure, 42.86% air, and 45.16% swabs. In KJK Szary, a high percentage of resistance to aztreonam and ceftazidime was found in bacteria isolated from air (respectively 23.53 and 11.76%) and swabs (19.15 and 17.02%), and also to cefalotin in strains isolated from swabs (10.64%). The percentage share of strains resistant to at least one or more tested antimicrobial agents in different environments was as follows: 28% manure, 41.18% air, and 38.30% swabs. In SKH Nielepice, no strains of *E. coli* were isolated from the air and the most probable reason for this was the non-stable horse keeping system in this facility. On the other hand, there was a very high percentage of strains isolated from swabs that were resistant to amoxicillin/clavulanic acid (21.43%), ampicillin (50%), cefalotin (35.71%), and ticarcillin (42.86%) and an increased level of resistance was observed in the case of aztreonam (14.29%), cefoxitin (14.29%), and cefazolin (14.29%). The percentage of strains resistant to at least one or more tested antimicrobial agents in different environments was as follows: 15.79% manure and 78.57% swabs. Maddox et al. (2012) found that on average 72.20% of *E. coli* isolated from horse manure exhibited resistance to at least one antibiotic. Ahmed et al. (2010) in their study conducted on 296 strains of *E. coli* isolated from 138 horses in northwest England observed the most frequent resistance to the following antibiotics: tetracycline 66.89%, ampicillin 64.53%, and ciprofloxacin 21.96%. High percentage of ampicillin-resistant *E. coli* strains was also observed in this study, but it was not observed in the case of tetracycline or ciprofloxacin. However, it should be remembered that the majority of resistant isolates in the study of Ahmed et al. (2010) were isolated from hospitalized horses, which certainly affected the increased percentage of resistance to the tested antimicrobials. In this study, horses were not hospitalized or treated pharmacologically with the use of antibiotics which would result in selective pressure and therefore affect the increase in the antibiotic resistance among bacteria (Dolejska et al. 2011). However, it should be remembered that the pharmacological therapy is not the only reason for the emergence of antimicrobial resistance. In the environment of horse riding centers, the source of resistant microorganisms may also involve people (Lenart-Boroń et al. 2017), horses that were not included in the study, but were given antibiotics (Bryan et al. 2010), and environmental elements, such as water (Alekshun and Levy 2007; Scott 2008; Lenart-Boroń 2017), fodder, and litter (Lenart-Boroń et al. 2016), or air (Heuer et al. 2011; Wolny-Koładka et al. 2014). Bryan et al. (2010) also found a high (20.40%) ampicillin resistance among *E. coli* isolated from horses. However, in their study, as much as 23.80% of *E. coli* strains were resistant to trimethoprim/ sulfamethoxazole, whereas the mean resistance to this antimicrobial agent of bacterial strains isolated from all three horse riding centers was 1.49%. Bryan et al. (2010) observed low resistance to ciprofloxacin (1.70%) and amoxicillin with clavulanic acid (0.50%) similarly as in the presented study, where the mean resistance to these agents was 0.32 and 4.20%, respectively. In studies by Maddox et al. (2012), mean resistance to ampicillin was 45.60% and to tetracycline—even 50.70%. The mean resistance to ciprofloxacin—similarly as in this study—was much lower (5.40%).

The ability to produce extended spectrum beta-lactamases was found in 34 isolates (13 OJK Pegaz, 16 KJK Szary, and 5 SKH Nielepice), whereas given the number of strains isolated from each horse riding center, it should be noted that they were most frequent in OJK Pegaz (20.31%). In OJK Pegaz, the ESBL-producing *E. coli* strains were most frequently isolated from the air (42.86%), in KJK Szary from swabs (19.15%), and in SKH Nielepice from manure (21.05%) (Table 2). Ahmed et al. (2010) found the ESBL mechanism in 5.74%, while Maddox et al. (2012) on average in 6.30% of *E. coli* isolates derived from horses. On the other hand, according to Hanberger et al. (2009), the prevalence of human ESBL-producing *E. coli* strains in Europe is 3.90% and varies considerably between countries.

The tested *E. coli* strains were mostly resistant to one or two antibiotics. Two MDR strains were found in OJK Pegaz (air and swab), and in KJK Szary, there were also two MDR strains (air and swab) and one XDR strain (swab) (Table 3). The MDR (multidrug-resistant) strains are resistant to at least three classes of antibiotics (Maddox et al. 2012), while XDR strains (extensively drug resistant) are resistant to all but two antibiotics used in the treatment of infections caused by the considered species (Magiorakos et al. 2012). Maddox et al. (2012) demonstrated that 37.60% of *E. coli* isolated from horse manure were multidrug resistant. Ahmed et al. (2010) found 38.80% of *E. coli* isolates to be MDR. Such high percentage of MDR strains compared with the presented study should be explained by the fact that 106 MDR isolates in the study by Ahmed et al. (2010) were derived from hospitalized horses. Similar studies on hospitalized horses and horses not subjected to pharmacological treatment were conducted by Bryan et al. (2010), who found the resistance to at least one antibiotic in 28.60% of *E. coli* strains and the MDR phenotype in 15.29% of isolates. Bryan et al. (2010) suggest that hospitalization and drug administration have significantly increased the resistance of horse-derived *E. coli*.

**Table 3:** Number of *E. coli* isolates with MDR and XDR phenotype and resistant to different numbers of antibiotics

	OJK Pegaz *n* = 64	KJK Szary *n* = 103	SKH Nielepice *n* = 33	Total *n* = 200	Mean
MDR	2	2	0	4	1.33
XDR	0	1	0	1	0.33
10	0	1	0	1	0.33
9	0	0	0	0	0
8	0	1	0	1	0.33
7	0	0	1	1	0.33
6	0	0	0	0	0
5	1	2	1	4	1.33
4	4	1	1	6	2
3	0	4	1	5	1.67
2	9	12	6	27	9
1	16	15	4	35	11.67
0	34	67	19	120	40

**Fig. 1:**
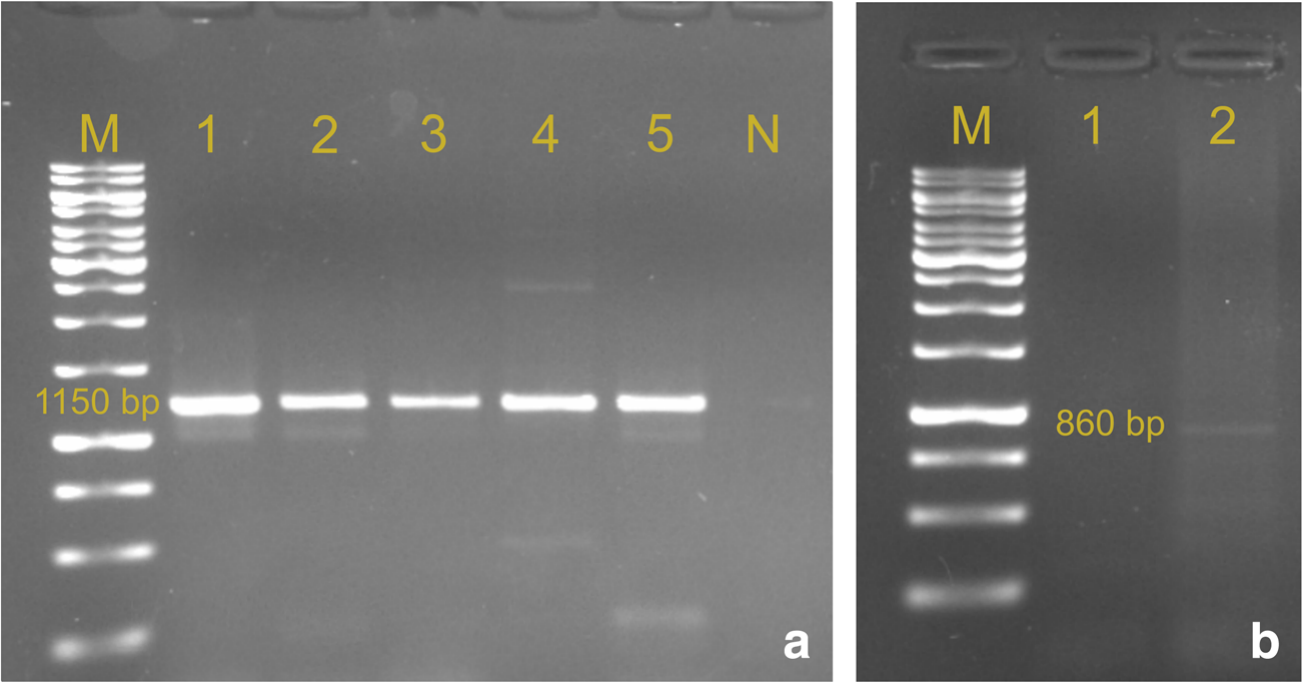
Results of PCR detection of ESBL-determining genes; **a** blaTEM, **b** blaCTXM-9. Lane M, DNA size marker GeneRuler 1 kb DNA Ladder (Thermo Scientific)

Among the extended-spectrum beta-lactamase genes, only *blaTEM* and *blaCTXM-9* were found in the analyzed *E. coli* strains, with no *blaCTXM-3*, *blaOXA*, and *blaSHV* (Table 2, Fig. 1). In addition, there were significant differences in the incidence of both genes. The *blaTEM* gene clearly dominated in all three horse riding centers, being most frequent in KJK Szary (53.4%). The *blaCTXM-9* gene in all cases occurred simultaneously with *blaTEM*, never individually. Ahmed et al. (2010) also found that the *blaTEM* gene was the most frequent, i.e., in as many as 91% *E. coli* strains isolated from horses, whereas the second ESBL-determining gene in their study—*blaSHV*—was detected in one isolate only. According to Bradford (2001), among the genes responsible for the production of beta-lactamases, the TEM family is most often detected in Gram-negative bacteria. Also, in *E. coli* and *K. pneumoniae*, genes from the TEM and SHV families are most frequently detected (Bradford 2001). According to Baraniak (2010) the TEM family of ESBL enzymes in Poland is most variable, as it is represented by at least 10 variants of β-lactamases, with eight identified only in Poland. TEM genes evolve as a result of subsequent point mutations, forming increasingly specialized enzymes, therefore allowing for adaptation of bacterial strains to various environments, where they get in contact with different β-lactam antibiotics. Baraniak (2010) in the epidemiological analysis of TEM-producing *E. coli* strains revealed a variety of epidemiological phenomena in Polish hospitals. In some of the tested facilities, outbreaks of clonal diseases were identified and a horizontal spread of plasmids carrying ESBL-conferring genes among strains of different *Enterobacteriaceae* species was demonstrated. The ampicillin resistance described in this study is correlated with the presence of the *blaTEM* gene (Ahmed et al. 2010). According to Brinas et al. (2002), *blaTEM* genes are often reported in ampicillin-resistant *E. coli* strains isolated both from animals and humans. Out of the 34 isolates, in which the ESBL mechanism was detected using the disk-diffusion method, 12 did not have any of the analyzed genes, which may be due to the fact that the mechanisms of resistance could be encoded by more than one gene, or by other genes that those examined in our study (Wolny-Koładka and Lenart-Boroń 2016). On the other hand, in 73 isolates, despite the presence of at least one of the ESBL-determining genes, the ESBL mechanism was not found in phenotypic tests. Similar observations have been reported by Wolny-Koładka and Lenart-Boroń (2016) where ESBL-determining genes were detected in 38% of *E. coli* strains, whereas in phenotypic tests this mechanism was not observed. Therefore, despite the discrepancies in the results between the disk-diffusion test and PCR, it is reasonable to detect this mechanism in both antibiogram tests and by using molecular analyzes. This is extremely important from an epidemiological point of view and will facilitate monitoring and control of potential infections (Gniadkowski et al. 2009). The results of the study confirm that the correlation between phenotypic and genetic tests is low. This may be due to the fact that the antimicrobial resistance is encoded by multiple genes, or that the genes present in *E. coli* cells were not expressed, in order to observe this mechanism in the phenotypic tests (Wolny-Koładka and Lenart-Boroń 2016).

**Fig. 2:**
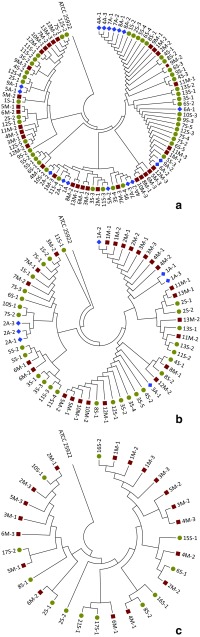
UPGMA dendrograms of *E. coli* strains isolated from various environments (A—air, M—manure, S—swabs) of three horse riding centers. **a** OJK Pegaz. **b** KJK Szary. **c** SKH Nielepice

The *χ*^2^ test confirmed statistically significant differences in the incidence of resistance (*p* < 0.05) to two antibiotics. The chi-square statistic values were 14.36 for ampicillin and 8.77 for ticarcillin. UPGMA dendrograms (Fig. 2a–c) were constructed in order to assess whether there are genetic similarities between *E. coli* strains isolated from various environments (A—air, M—manure, S—swabs) within individual horse riding centers. The strains were differentiated based on the rep-PCR (BOX-PCR) reaction, which allowed to assess the heterogeneity of the analyzed groups of *E. coli* strains. The analysis comprised 182 strains where the BOX-PCR bands were detected and a reference strain ATCC 25922. The figures show that the analyzed environmental strains in each of the studied facilities are clearly different from the reference strain (ATCC 25922). As shown in Fig. 2a–c, the bacterial isolates are characterized by large genetic diversity and even strains originating from the same horse were grouped in different clusters (i.e., strains marked as 2S-1 and 2S-2 and 2M-1 to 2M-3 were isolated from swabs and manure from the same animal). This observation is also supported by the results of AMOVA analysis (Table 4), as the variation within populations is very high and covers 93.59% of overall variation. On the other hand, haplotype analysis revealed the presence of 59 haplotypes, with 32 being carried by one strain only. However, there is one haplotype characteristic for as many as 27 isolates (13 from KJK Szary, 5 from SKH Nielepice, and 9 from the OJK Pegaz horse riding center, Table 5). This haplotype is represented—among others—by two strains from KJK Szary of which one was isolated from manure and the second one from swab of the same horse. The same situation occurs for 6 other strains in KJK Szary and 12 in OJK Pegaz, whereas in Nielepice we did not observe any common haplotype between strains isolated from swabs and manure collected from the same horse. The most haplotype rich facility was KJK Szary, where 0.52 haplotype per isolate was observed. Almost the same ratio was detected for SKH Nielepice (0.5 haplotype per isolate), and the lowest value was recorded for OJK Pegaz center, with only 0.26 haplotype per isolate. This indicates that the highest variation among *E. coli* strains, shown by the haplotype abundance, was observed for the horse riding center where the largest number of horses are kept. Also, as expected, almost the same high variation was typical of the free-range facility (SKH Nielepice), where also no “shared” haplotypes were detected (Table 5). On the other hand, *E. coli* strains isolated from the horse riding center Pegaz, which was characterized by the smallest haplotype richness, also showed the highest percentage of ESBL-carrying strains. It may indicate that the type of the horse keeping (stable vs. free-range) affects the biodiversity of their microflora and the probability of antimicrobial resistance spreading, therefore affecting their health and health of their users. These results are similar to those obtained by Lenart-Boroń et al. (2016), in the study on antimicrobial resistance and molecular diversity of *E. coli* isolated from chicken feces. This research revealed that the strains isolated from an organic farm were most variable, and characterized by the lowest level of resistance to several antimicrobial agents, among strains from five different farms.

**Table 4:** The results of AMOVA analysis for 183 isolates grouped into three populations (horse riding centers)

Source of variation	Sum of squares	Variance components	Percentage of variation
Among populations	15.876	0.10230	6.41
Within populations	267.260	1.49307	93.59
Total	283.137	1.59537	100
Fixation index—F_st_	0.06412

**Table 5:** Characteristics of most frequent^a^ haplotypes within strains isolated from the analyzed horse riding centers

Haplotype	No. of isolates	Detected in (environment)			Detected in (facility)		
		A (*n* = 24)	M (*n* = 75)	S (*n* = 83)	N (*n* = 30)	P (*n* = 58)	S (*n* = 94)
Ec_1	27	3	15	9	5	9	13
Ec_2	17	6	4	7	1	2	14
Ec_3	14	1	6	7	0	11	3
Ec_4	10	2	4	4	3	5	2
Ec_5	8	0	5	3	3	4	1
Ec_6	8	0	5	3	2	4	2
Ec_7	7	0	6	1	6	0	1
Ec_8	7	0	3	4	0	7	0
Ec_9	6	0	2	4	0	4	2
Ec_10	5	0	2	3	1	0	4
Ec_11	4	3	0	1	1	3	0
Ec_12	4	0	0	4	2	2	0
Ec_13	3	1	1	1	0	0	3
Ec_14	3	0	2	1	1	0	2
Ec_15	3	0	2	1	0	0	3
Ec_16	3	2	0	1	1	2	0

^a^Only haplotypes specific for three or more isolates are presented

## Conclusion

The conducted study allowed for the isolation, identification, and assessment of antimicrobial resistance profile of 200 *E. coli* strains from the environment of three horse riding centers, differing in the type of horse keeping. Among the collected strains, many were resistant to the tested antibiotics, including the presence of bacteria presenting the MDR and XDR phenotypes. It indicates that the studied horses are a source of antimicrobial-resistant *E. coli*. Analysis of genetic diversity demonstrated a high variation among the analyzed strains, whereas its degree differed between the studied facilities, indicating that the type of horse keeping (stable vs. free-range) affects the genetic diversity of the commensal microflora, represented by the species of *E. coli*. The ability to produce extended-spectrum beta-lactamases has been demonstrated in the disk-diffusion test, as well as by detecting the ESBL-encoding genes, and the *blaTEM* gene was the most abundant among the ESBL-determining genes. At the same time, it should be remembered that detection of the ESBL mechanism by phenotypic methods may produce false negative results. Therefore, given the discrepancies between the results of phenotypic and molecular tests, it is reasonable to conduct further studies aimed at identifying the risk factors for the spread of drug resistance among horses. This is particularly important because horses are among the components of zoonotic transmission of antibiotic-resistant bacteria and can be both recipients and the reservoir of the resistance genes, which poses a major threat to public health.
